# Mental health and well-being of students at TU Dresden

**DOI:** 10.1093/eurpub/ckac131.488

**Published:** 2022-10-25

**Authors:** M Girbig, L Huebner, D Kaempf, T Lesener, B Gusy, A Seidler

**Affiliations:** 1 Institute and Policlinic for Occupational Polyclinic for Occupational and Social Medicine, TU Dresden, Dresden, Germany; 2 Department of Education and Psychology, FU Berlin, Berlin, Germany

## Abstract

**Background:**

University life can be a particularly challenging phase in the development of young adults. Current research shows that mental disorders occur more frequently in students compared to workers of the same age. The coronavirus pandemic has exacerbated the problem. The TUDo! Study, done in cooperation with the FU Berlin, aimed to assess the health status of students at TU Dresden, with a focus on mental health.

**Methods:**

In 2020/2021 students at TU Dresden (excluding students in the medical school) completed an online-based questionnaire. Validated questionnaires, e.g. the PHQ 4 (depressive disorder and generalized anxiety disorder) and the ERI student (student gratification crisis) were used. We evaluated descriptively and analytically, according to the questionnaire-specific specifications.

**Results:**

A total of 2,683 students (12.3%) at the TU Dresden took part in the survey. The majority of study participants were female (n = 1,507; 56.7%) and had an average age of 22.9 years (SD = 4.3). 32.8% (n = 856/2,611) of the participating students reported a depressive syndrome and 32.5% (n = 848/2,612) a generalized anxiety disorder. Almost half of participating students (40,0%; n = 515/1,310) indicated an imbalance between effort (E) and reward (R) (ER ratio>1). 51.23% (n = 693/1,50) of respondents reported a decline of mental well-being because of the coronavirus pandemic.

**Discussion:**

Unlike similar studies, this study shows that TU Dresden students were particularly affected with regards to perceived psychological stress and complaints. The existing differences seem to be partly due to the coronavirus pandemic. These results indicate that universities should regularly check their studying conditions and provide appropriate preventive measures.

**Key messages:**

• Students are at higher risk than workers of the same age for mental health problems.

• Students reported a decline in mental well-being due to the coronavirus pandemic.

